# Unraveling the Protein Network of Tomato Fruit in Response to Necrotrophic Phytopathogenic *Rhizopus nigricans*

**DOI:** 10.1371/journal.pone.0073034

**Published:** 2013-09-02

**Authors:** Xiaoqi Pan, Benzhong Zhu, Yunbo Luo, Daqi Fu

**Affiliations:** The College of Food Science and Nutritional Engineering, China Agricultural University, Beijing, PR China; Swiss Federal Institute of Technology (ETH Zurich), Switzerland

## Abstract

Plants are endowed with a sophisticated defense mechanism that gives signals to plant cells about the immediate danger from surroundings and protects them from pathogen invasion. In the search for the particular proteins involved in fruit defense responses, we report here a comparative analysis of tomato fruit (*Solanum lycopersicum* cv. Ailsa Craig) infected by *Rhizopus nigricans* Ehrenb, which is a significant contributor to postharvest rot disease in fresh tomato fruits. In total, four hundred forty-five tomato proteins were detected in common between the non-infected group and infected tomato fruit of mature green. Forty-nine differentially expressed spots in 2-D gels were identified, and were sorted into fifteen functional groups. Most of these proteins participate directly in the stress response process, while others were found to be involved in several equally important biological processes: protein metabolic process, carbohydrate metabolic process, ethylene biosynthesis, and cell death and so on. These responses occur in different cellular components, both intra- and extracellular spaces. The differentially expressed proteins were integrated into several pathways to show the regulation style existing in tomato fruit host. The composition of the collected proteins populations and the putative functions of the identified proteins argue for their roles in pathogen-plant interactions. Collectively results provide evidence that several regulatory pathways contribute to the resistance of tomato fruit to pathogen.

## Introduction

*Rhizopus nigricans* Ehrenb is a major necrotrophic phytopathogenic fungus that causes serious decay on fruit during development and postharvest storage [1]. Because of the ubiquity of fungus and the easily spread of spores, soft fruits being of abundant nutrition are much more susceptible. Due to the markedly shorten of storage life and serious economic loss, disease caused by *R. nigricans*, pre- and postharvest, has become a hot topic for these years in research work.

Because of the great achievement in genomics, as well as availability of various mutants, tomato fruits are deemed globally as an ideal material in many plant biological and physiological researches, including seed germination [2], fruit ripening and development [3], abiotic stresses [4] and biotic stress [5]. The analysis of plant responses to biotic stress in terms of biochemical and molecular changes provides unique information to dissect the mechanisms that plants are endowed to resist diseases. Due to the high input of phytochemicals, environmental concerns are pushing modern agriculture to fight against pathogens by exploiting the natural resistance of the species. Thus the identification of molecular resistance mechanisms becomes a central issue [6].

A substantial number of previous studies demonstrated that resistance to pathogen infection is accomplished through a diverse array of antimicrobial chemicals and PR proteins [7], [8]. However, the defense response of plant to pathogen is such a sophisticated mechanism that it needs an integrated network of a serious of elements and pathways to clarify this process. Proteomics has emerged as a high throughput and powerful tool to investigate the important biological phenomenon to uncover the involvement of sets of gene products. To date there have been several studies concerning to proteome analysis in pathogen defense of tomato fruit. Shah et al. (2012) [9] carried out a proteomic analysis of tomato fruit infected by *Botrytis cinerea*, which is another kind of necrotrophic fungi causing deterioration of fruits. They identified 119 and 456 tomato proteins from infected mature green and red ripe tomato fruit, respectively. And 25 and 33 of these proteins were observed as differentially expressed proteins, providing us valuable information in this research field.

Here, we adopted high-resolution two-dimensional (2D) electrophoresis combined with complementary molecular and physiological techniques to identify target genes in terms of host resistance to pathogen. And the proteomic data were integrated into a network containing several metabolic or regulatory pathways in the form of protein-protein interaction (PPI) to gain a systematic and broader range of elucidation. It is an efficient way to predict plant defense mechanism or regulatory style. We identified 458 and 466 proteins in non-inoculated (*N-Ino*) and inoculated (*Ino*) mature green tomato fruit, respectively. Forty nine of these proteins were differentially displayed. These proteins represented diverse of biological processes including biotic and abiotic stress responses, metabolic process, ethylene biosynthesis, hydrogen peroxide catabolic processes and cell death. Our study also provides evidence that pathogen-invasion accelerates fruit ripening process, and the Calvin cycle switches from normal metabolism to defense in response to *R. nigricans*. Moreover, our study provides complementary information that helps us to dissect how plants make response to pathogen through host metabolic regulation and gene expression.

## Materials and Methods

### Plant Material

Tomato seeds were planted in a mixture of potting soil:vermiculite (3∶1 [v/v]), and seedlings were transplanted to commercial tomato-cultivated soil when they have 3–4 true leaves. All plants were grown in typical greenhouse and flowers were tagged one day after anthesis (DPA). Fruits were harvested at MG (mature green, 34 DPA), BK (break, 38 DPA), PK (pink, 42 DPA) and RR (red ripe, 48 DPA) stages of fruit ripening. Ripening stages were also confirmed by visual analysis of color, size, shape as described previously [10]. Fifteen fruit were identified and collected from 5–6 plants, sterilized by immersion in 10% bleach for 5 min followed by four rinses with distilled water.

### Inoculation with *Rhizopus nigricans* Ehrenb

*R. nigricans* strain was provided by Dr. Sheng (Renmin University of China) and was originally isolated from tomato fruit. Fungal strain was routinely cultured on potato dextrose agar (PDA) at 26°C, and spores harvested from 10-day-old cultures with sterile water containing 0.01% (v/v) Tween-80. After removing mycelial debris by filtration, spores were quantified with hemacytometer and diluted to 800 conidia µl^−1^ for inoculation. Fruit were inoculated with 5 µL of inoculum at each site according to the method described by Pan et al. (2013) [1]. The fruit in negative control group was wounded and treated with 5 µL of sterile water at each site. Susceptibility and severity were determined daily as disease incidence and the diameter of the macerating lesion according to the following formula:
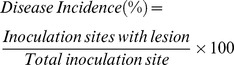



Fruit tissue was harvested from appropriate 0.5–1 cm around the wounded sites, pooled from fifteen lesions per pool (three pools per line), and stored at −80°C until further analysis.

### Population Studies of *R. nigricans* in Fruit Wounds

Wounded tissue was collected each day after inoculation at 26°C for 4 days, and *R. nigricans* was recovered from the collected wounded according to Zhang et al. (2008) [11]. Serial 10-fold dilutions were made and 0.1 mL of each dilution was plated on PDA. The plates were incubated at 26°C for 2 days and the colonies counted. Population densities of *R. nigricans* were expressed as log_10_ CFU per wound site. Three single fruit were involved in per treatment, and the experiment consists of three replications.

### Two-dimensional (2D) Gel Electrophoresis of Total Protein

Total proteins were extracted from tomato fruit at 48 hours post-inoculation (HPI) as described by Saravanan and Rose (2004) [12], dissolved in IEF buffer and the protein concentration determined by Bradford assay (1976) [13] using bovine serum albumin as a standard. IPG strips (13 cm pH 4–7, Bio-Rad ReadyStrip, Bio-Rad) were rehydrated overnight with 200 µL of IEF buffer containing 500 µg of total proteins. Isoelectric focusing (IEF) was performed on an Ettan IPGphor unit (GE Healthcare Bio-Sciences AB, Uppsala, Sweden) at 20°C, applying the following program: a linear increase from 0–500 V over 1 h, 500 V to 1000 V over 1 h, 1000 V to 8000 V over 2∶30 h and then held at 8000 V for 0∶55 h. After focusing, the proteins were reduced and alkylated followed by equiliberation in a buffer containing 6 M urea, 30% w/v glycerol, 2% SDS, and 50 mM Tris-HCl, pH 8.8. The proteins on the equiliberated strip were separated in the second dimention on a 15% SDS-PAGE gels. 2-DE gels, after electrophoresis, were stained with Coomassie Brilliant Blue (CBB) R-250 and scanned by a flatbed scanner (GE Healthcare Bio-Sciences AB) followed by spot detection and matching between gels automatically. Gel pictures were stored in TIF. Proteins whose expression was significantly different between the control and inoculated tissues were identified by statistical analysis (Student’s *t*-test) and the change-folds of these proteins were also calculated.

### Protein Digestion, MS Analysis and Identification

Individual protein spots were excised from a gel slab using a clean scalpel, placed into 1.5 mL eppendorf tubes. Gel pieces were dehydrated in 100 µL of 50 mM NH_4_HCO_3_/acetonitrile (1∶1) for 20 min at 37°C, followed by a dehydration step in 50 µL acetonitrile for 10 min. Prior to addition of sequencing grade porcine trypsin (Promega, Madison, USA) at 10 ng µL^−1^, gel pieces were dried down in a vacuum centrifuge for 5 min. Then samples dissolved in trypsin solution were placed into an air circulation thermostat and incubated overnight at 37°C. Upon in-gel digestion, gel pieces were saturated with 50 µL extraction buffer of 0.1% TFA/ACN, 0.1% TFA/H_2_O and 0.1% TFA/50%ACN for once respectively with incubating at 37°C in shaker for 10 min each time. Supernatants were then collected, pooled together and dried down in a vacuum centrifuge. For LC-MS/MS analyses, 0.05% trifluoroacetic acid (TFA) (10 µL) was added into the dried tubes, incubated for 2 min and vortexed for 10 min at 7 g.

LC-MS/MS was performed on a Q-TOF micro mass spectrometer equipped with CapLC high performance liquid chromatography system (Waters Ltd., Manchester, U.K.) to establish peptide identity as described by Qin et al. (2012) [14]. The generated pick lists were uploaded to Mascot search program (http://www.matrixscience.com) using NCBI non-redundant protein databases. “Viridiplantae”, “Solanaceae”, “Tomato” and “fungi” were selected as the taxonomic category for all MS results. MASCOT identifications of proteins were considered confident if: 1) matching scores above the threshold (see Table 1), 2) number of matched peptides ≥4/2 and sequence coverage ≥10/4% for Q-TOF. When there were several matching results, the one that of highest score were listed in Table 1.

**Table 1:** Identified Tomato Proteins Involved in Pathogen-Tomato Interaction.

Spot	Protein name	Accession Number	Organism	Protein score a	Mr (kDa)	PI	NUMP b	Fold ^c^
Response to stress								
1	17.7 kD class I small heat shock protein	gi|4836469	Solanum lycopersicum	174(65)	17724	5.84	11	2.839
2	heat shock protein 70	gi|350537379	Solanum lycopersicum	380(53)	74243.5	5.41	20	0.297
17	small heat shock protein	gi|8918494	Solanum lycopersicum	349(53)	21466.3	5.89	14	2.777
18	pathogenesis-related protein PR P23	gi|19315	Solanum lycopersicum	163(53)	25111.5	6.13	8	3.102
23	beta-1,3-glucanase	gi|170380	Solanum lycopersicum	71(53)	37572	6.6	5	5.860
30	acidic 26 kDa endochitinase precursor	gi|350534584	Solanum lycopersicum	468(53)	27614.3	5.93	15	2.785
36	class I small heat shock protein 17.6	gi|349591295	Solanum lycopersicum	388(53)	17634	5.84	19	0.067
41	cytosolic class II small heat shock protein HCT2	gi|3639075	Solanum lycopersicum	116(53)	17321.9	6.75	8	3.216
40	pathogenesis-related protein PR P23	gi|19315	Solanum lycopersicum	134(53)	25111.5	6.13	13	7.594
42	STH-2 protein	gi|169576	Solanum tuberosum	346(59)	17283.8	5.66	20	12.71
44	cytosolic ascorbate peroxidase 1	gi|73761751	Solanum lycopersicum	142(53)	27390.8	5.61	21	3.853
**Protein metabolic process**								
11	RAD23-like	gi|77745475	Solanum tuberosum	260(59)	40607.5	4.71	12	0.361
22	26S protease regulatory subunit 6A homolog	gi|1729860	Solanum lycopersicum	261(59)	47475.4	4.94	24	0.396
33	Skp1-like protein 3	gi|82470777	Petunia integrifolia subsp. inflata	67(59)	17574.7	4.61	12	0.452
39	proteasome-like protein alpha subunit	gi|77999303	Solanum tuberosum	86(59)	27121.6	5.63	21	3.634
43	Hop-interacting protein THI111	gi|365222920	Solanum lycopersicum	121(53)	44676.5	5.1	9	0.219
**Carbohydrate metabolic process**								
14	alpha-mannosidase	gi|301176645	Solanum lycopersicum	179(53)	116408.8	6.18	13	6.958
20	alpha-galactosidase	gi|10312171	Solanum lycopersicum	391(53)	41841	4.95	27	0.444
26	chloroplast sedoheptulose-1,7-bisphosphatase	gi|238563983	Solanum lycopersicum	433(53)	42560.6	6.07	32	0.294
46	glyceraldehyde 3-phosphate dehydrogenase	gi|2078298	Solanum lycopersicum	298(53)	31926.2	5.93	14	0.196
**Ethylene biosynthesis**								
4	1-aminocyclopropane-1-carboxylate oxidase homolog	gi|119640	Solanum lycopersicum	72(59)	41050.5	5.61	10	3.252
6	1-aminocyclopropane-1-carboxylic acid oxidase	gi|308390267	Solanum lycopersicum	274(53)	35790	5.13	33	3.002
7	Peptide methionine sulfoxide reductase	gi|1709692	Solanum lycopersicum	436(53)	21904.6	6.1	17	3.397
13	1-aminocyclopropane-1-carboxylate oxidase homolog	gi|119640	Solanum lycopersicum	122(65)	41050.5	5.61	13	3.770
15	1-aminocyclopropane-1-carboxylate oxidase homolog	gi|119640	Solanum lycopersicum	365(65)	41050.5	5.61	22	3.440
**Hydrogen peroxide catabolic process**								
12	Suberization-associated anionic peroxidase 2	gi|129811	Solanum lycopersicum	124(53)	38523	4.57	6	0.120
19	catalase	gi|170398	Solanum lycopersicum	331(53)	56470	6.56	23	0.457
45	Catalase isozyme 2	gi|1705616	Solanum lycopersicum	79(59)	56415	6.56	11	3.834
**Glycolysis**								
5	enolase	gi|1161573	Solanum lycopersicum	177(53)	35073.1	6.29	19	2.360
8	ethylene-responsive enolase	gi|5669648	Solanum lycopersicum	77(53)	7756.8	4.75	2	2.042
**Transport activity**								
9	ATP synthase CF1 beta chain	gi|89280642	Solanum lycopersicum	1120(53)	53433.9	5.28	42	2.319
34	ATP synthase F1 subunit 1	gi|316996023	Nicotiana tabacum	144(59)	55289	5.84	12	0.070
**Calcium ion binding**								
27	Calmodulin	gi|2388889	Solanum lycopersicum	80(65)	13307.3	4.1	6	0.256
**Cell death**								
28	14-3-3 protein 9	gi|15637116	Solanum lycopersicum	327(53)	28902.3	4.71	25	2.330
**Purine ribonucleoside salvage**								
31	adenine phosphoribosyltransferase-like	gi|82621166	Solanum tuberosum	71(59)	19821.6	5.17	5	2.926
**Terpenoid biosynthetic process**								
37	ISPH protein	gi|262176919	Solanum lycopersicum	90(53)	51862.2	5.38	23	2.574
**Purine catabolic process**								
38	chloroplast elongation factor TuB (EF-TuB)	gi|218312	Nicotiana sylvestris	105(59)	46672	5.7	21	3.416
**virus-host interaction**								
21	viral envelope protein	gi|1644306	Nicotiana tabacum	76(59)	6754.4	4.72	4	3.020
**Others**								
3	plastid transketolase	gi|194396261	Nicotiana tabacum	103(59)	80051.4	6.16	11	0.197
10	hypothetical protein	gi|2213425	Citrus x paradisi	95(65)	32622.6	5.46	6	2.378
16	formate dehydrogenase	gi|56562181	Solanum lycopersicum	272(53)	42066.6	6.87	34	0.375
24	Capsid protein	gi|116818	Solanum lycopersicum	539(53)	17734.9	4.85	7	0.070
25	BTF3-like transcription factor	gi|83584406	Solanum lycopersicum	233(53)	17397.1	6.85	11	0.325
29	At2g16900/F12A24.8	gi|14532492	Arabidopsis thaliana	74(65)	42969.1	5.44	10	0.058
32	alcohol dehydrogenase-2	gi|170368	Solanum lycopersicum	246(53)	41013.8	6.03	21	0.045
35	Capsid protein	gi|10719960	Solanum lycopersicum	393(53)	17750.9	4.85	11	0.017

a Mascot matching score (the threshold value).

b NUMP is the abbreviation of number of unique matched peptides.

C Changed expression level in inoculated tissue compared with that in non-inoculated ones.

### Protein Functional Annotation

Differentially displayed proteins were classified according to annotations from the UniProt knowledge base (Swiss-Prot/TrEMBL, http://www.uniprot.org/) and the GO database (http://www.geneontology.org/). The biological process and molecular function were elucidated according to David database (http://david.abcc.ncifcrf.gov/) and GOTERM and PANTHER, respectively [15]. Cellular components were elucidated according to GOTERM. The KEGG database (http://www.genome.jp/kegg/)and PANTHER_PATHWAY was used complementing to elucidate the pathways of differentially expressed protein. InParanoid database analyses (version 7) (http://inparanoid.sbc.su.se/cgi-bin/index.cgi) were performed to identify the eukaryotic orthologous proteins of differentially expressed tomato proteins by sequence blast. STRING database (version 9.0) (http://string-db.org/) was used to predict and visualize the PPI networks among differentially expressed proteins.

### RNA Extraction and qRT-PCR

Fresh fruit material of tomato within a 1.0-cm radius around the inoculation or wounded sites was collected, and each biological replicate consisted of an independent pool of samples from six different fruit. Total RNA was extracted as follows: two grams of tissue sample pool were grounded to a fine powder in liquid nitrogen, and 4 mL of extraction buffer (containing 100 mM Tris-HCl, 25 mM EDTA, 75 mM NaCl, 1% SDS and 10 mM β-mercaptoethanol) was added. The extraction mixture was mixed on a vortex, followed by the addition of 2.4 mL chloroform:isoamyl alcohol (49∶1), and centrifugation at 4°C at 10,000 g for 15 min. The aqueous phase was removed and RNA was precipitated with equal volume of isopropanol and 350 µL of NaAc-HAc for 1 h at −20°C. The precipitated RNA was pelleted by centrifugation for 15 min at 4°C and 10,000 g, followed by washing with 75% ethanol. After air drying the RNA was resuspended in 50 µL DEPC H_2_O. The RNA pellet was treated with DNase (RNase-Free; Promega) followed by extraction with chloroform:isoamyl alcohol (49∶1). The RNA concentration and purity were measured using a NanoDrop 1000 Spectrophotometer (Thermo Scientific). The RNA integrity was checked by agarose gel electrophoresis.

cDNA was synthesized using reverse transcription System (Promega, Madison, WI, USA) as described in Pan et al. (2013) [1].

The quantitative Real-time PCR was performed using SYBR Green PCR Master Mix with a BIO-RAD Real-Time PCR System (BIO-RAD CFX96, USA). Gene-specific qRT-PCR primer pairs were listed in Table 1, and the PCR condition was as follows: 95°C for 10 min, followed by 40 cycles of 95°C for 15 s and 60°C for 30 s. Fluorescence changes of SYBR Green were monitored automatically in every cycle, and the threshold cycle (Ct) over the background was calculated for each reaction. Samples were normalized using *18* *S* rRNA (SGN-U581385) and the relative expression levels were measured using the 2^(−ΔCt)^ analysis method [16]. Oligonucleotide primers used in this study are listed in Table 2. The RT-PCR data presented are representative of three independent experiments.

**Table 2:** Oligonucleotide primers used in this study.

Gene name	Forward primer 5′–3′	Reverse primer 5′–3′
***E4***	TCAGCCGGGTCTGGAGTTT	CTCCTCCAACCCTCTGGAAAG
***E8***	CACGTGCTTCTTCGGTGAAA	GGTTGCGCGATATTTTGGA
***TFT9***	TGCCACTGCTAGTTCAGATCTTG	CTCGGGTGAATTCAGAATCTCAT
***Hsp70***	TTTGCCCAAAGATGAGGTTGA	TGCTTGATTCTTCGCGTCTATG
***Hsp17.6***	AAGCTCATGTGTTCAAGGCTGAT	TTCCTCTCTCCGCTGATCTGA
***alpha-Gal***	CGCAGGGAAATATGGTTGCTA	TCCTTTGCCGTGAACATAATCTG
***18*** **** ***S***	AGCCTGAGAAACGGCTACCA	TGTCACTACCTCCCCGTGTCA

### Enzyme Activity Assay

The activity of peroxidase (POD) was assayed according to Lurie et al. (1997) [17] with little modification. The reaction mixture contained 0.5 mL of crude extract, 2.5 mL of 25 mM guaiacol and 0.2 mL of 250 mM hydrogen peroxide. The reaction was allowed to proceed for 4 min with OD measurements taken every 60 s, beginning 1 min after adding the crude extract to the substrate.

CAT (catalase) activity was assayed using a method described by Havir and MuHale (1987) [18] with little modification. The reaction mixture contained 3 mL of 20 mM H_2_O_2_ in 50 mM acetic acid buffer (pH 7.5), and 100 µL of enzyme aliquot. The assay was carried out by the measure offing the absorbance at 240 nm every 30 seconds interval using an ultraviolet spectrophotometer (UNICO, American). All enzyme assays were performed in triplicate.

### Statistical Analysis

Microsoft Excel 2003 and SPSS 13.0 (SPSS Inc., Chicago, IL, USA) were used for the statistical analyses. Data were subjected to an analysis of variance (ANOVA), and a comparison of means was carried out by Student’s *t*-test. Differences were considered to be significant for p<0.05.

## Results and Discussion

### Positive Correlation between Ripening and Susceptibility of Tomato Fruit to *R. nigricans ehrenb*

**Figure 1:**
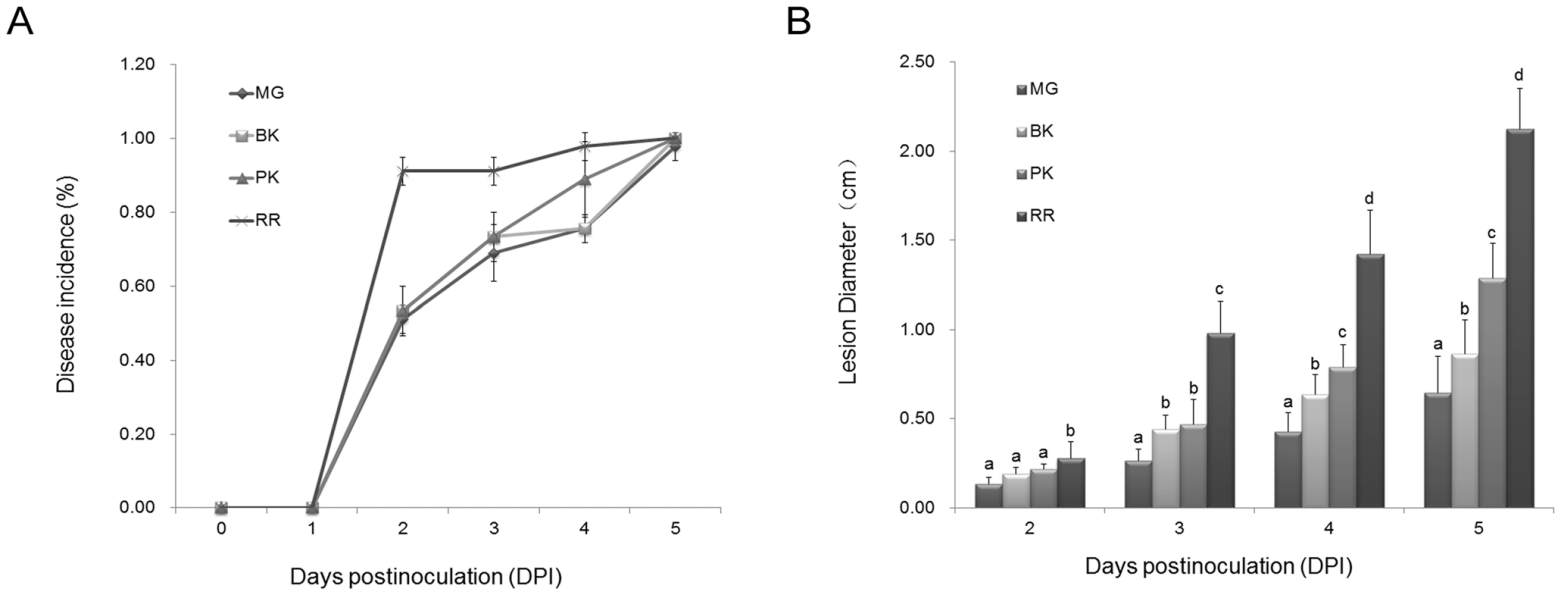
Tomato fruit ripening enhances both the development and severity of disease. A) The disease pattern was assessed by disease incidence (% total fruit). B) Disease severity by the lesion diameter (cm). Tomato fruits of MG, BK, PK and RR were inoculated with either 5 µL of inoculum containing 800 conidia µl^−1^ or 5 µL of water. Fruits were quantified each day post-inoculation (DPI) for incidence of disease development and the size disease lesion. Values show the mean±SE. The data were pooled from three independent experiments, with a total of 45 tomato fruit per treatment. Significant differences among fruit of different ripening stage were determined by Student’s *t*-test using SPSS 13.0 software and indicated by different letters (at P<0.05)).

**Figure 2:**
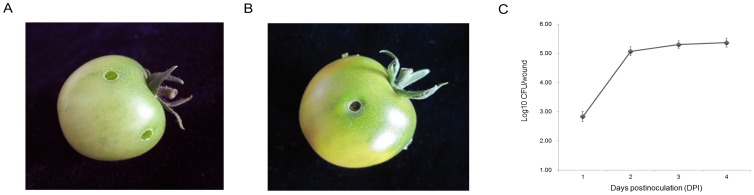
Tomato fruit infected by *R. nigricans* at the MG stages. Non-inoculated (A) and inoculated fruit (B) were photographed at 2 DPI. (C) Population dynamics of *R. nigricans* in wounds of tomato fruit. The Population density, log10 CFU per wound±SE, of *R. nigricans* was measured each day post-inoculation as described in Material and Methods. Data were pooled from three independent experiments, with a total of 9 wounded sites per treatment.

Generally, postharvest necrotrophic fungus can infect tomato fruits of different ripening stages. To examine the relative resistance of tomato fruit to *R. nigricans*, fruits of MG, BK, PK and RR were inoculated with either 50 µL of inoculum containing 800 conidia µL^−1^ or 5 µL of water. As shown in figure 1, the disease incidence and lesion development increased along with fruit ripening. The RR fruits exhibited the most serious disease development, however, MG tomato fruit showed the least disease incidence and tissue maceration on infected area. To unravel the underlying mechanism for the relatively strong resistance of MG fruit, fruits at 2 DPI (Fig. 2) were examined for differential protein expression, with or without *R. nigricans* infection.

### Protein Profiling of Tomato Fruit Infected by *R. nigricans*

**Figure 3:**
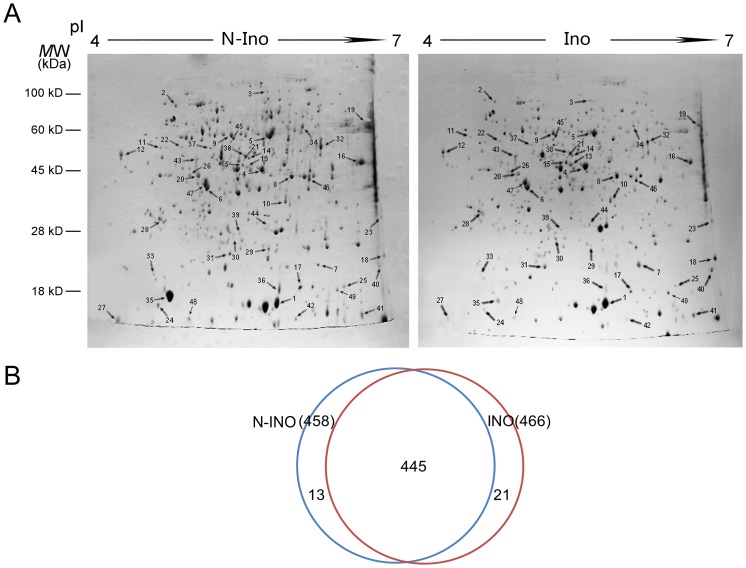
Changes in protein expression profile in inoculated fruit reveal the potential regulatory mechanism of biotic defense. (A) Proteomic identification of differentially expressed proteins between WT and Inoculated fruits at MG ripening stages. Protein extracts of 500 µL were analyzed in first dimension (pH 4–7 linear IPG, 13 cm) and by SDS-PAGE (15% T) in the second dimension, followed by visualization in Coomassie blue staining. Numbers indicate proteins that differentially expressed. (B) Area-proportional Venn diagrams depict the overlap of identified tomato proteins between WT and Inoculated fruit.

To acquire a comprehensive knowledge of fruit defense mechanism, we did a protein profiling for the MG fruit infected by *R. nigricans*. A total of 458 and 466 protein spots, from the *N-Ino* and *Ino* tissues respectively, were obtained by 2D gel electrophoresis separation. Spots that were very faint or were of undefined shapes and areas were not considered (Fig. 3A). Venn diagram (Fig. 3B) depicts the detailed information of detected numbers from *N-Ino* and *Ino* fruit sample, and 445 proteins (96.33%) were in common between the two tissues with different treatment. Among which 49 protein spots exhibited more than two-fold changes (p<0.05) in *Ino* fruit compared with the *N-Ino*. Each of these spots was excised from the gels and the identities of 46 spots were established by Q-TOF MS/MS analysis followed by database searching (Mascot searching program). Table 1 summarizes the detail information of these identified proteins according to their functional categories. Figure S1 shows the spectra details of proteins that scored below the threshold.

### Classification of Differentially Expressed Proteins

**Figure 4:**
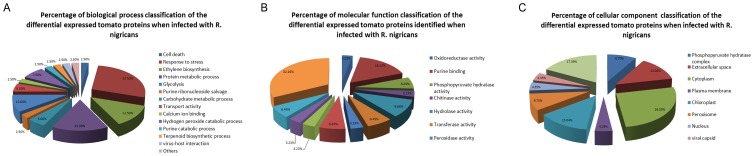
Functional classification of proteins exhibiting significant differential expression in *R.* *nigricans* inoculated compared to non-inoculated pericarp. Proteins were classified according to GO biological processes (A), molecular functions (B) and cellular components (C) by the David database.

Gene Ontology (GO) database and UniProt knowledge base were used to categorize the proteins from the *R. nigricans*-tomato interaction. The functional assignments of the identified tomato proteins with biological processes, molecular functions and cellular components are shown in Fig. 4.

**Figure 5:**
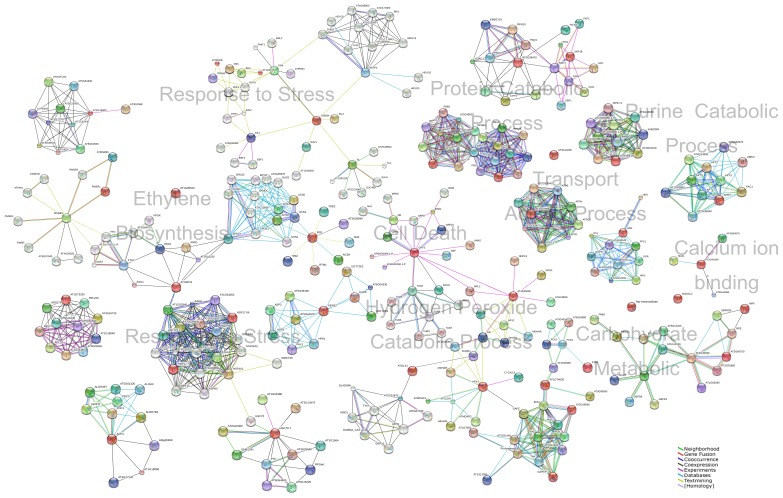
The representative protein-protein interaction (PPI) networks based on proteins differentially expressed in response to *R.* *nigricans* infection. The PPI networks were constructed by using the InParanoid database and String database according to GO and PANTHER biological processes, molecular functions, or KEGG pathways were integrated with the String database. The gene names of corresponding proteins are displayed in the networks. The red nodes represent those proteins that are differentially expressed between the non-inoculated and inoculated tomato fruit.

The biological processes that the differentially expressed proteins were involved in response to stress (27.50%), protein metabolic process (15.00%), ethylene biosynthesis (12.50%), carbohydrate metabolic process (10.00%), hydrogen peroxide catabolic process (7.50%), glycolysis (5.00%), cell death (2.50%), purine ribonucleoside salvage (2.50%), purine catabolic process (2.50%), terpenoid biosynthetic process (2.50%), virus-host interaction (2.50%) and others (2.50%) (Fig. 4A). Molecular function of these proteins was classified into 4 main categories: binding activity, enzyme activity, structural molecule activity and miscellaneous (Fig. 4B). Overall, the enzymes appeared were associated with oxidoreductase (12.90%) and hydrolase (9.68%). Moreover, according to subcellular classification, 30.43% (including one more in miscellaneous category), 13.04% and 13.04% of the differentially expressed proteins exist in cytoplasm, extracellular space and chloroplast, respectively (Fig. 4C). Because there is no comprehensive information of tomato PPI available to date, the InParanoid database were performed to identify the eukaryotic orthologous proteins of differentially expressed tomato proteins (Table S1). The PPI networks were constructed according to the String database, and the representative PPI network was profiled by integrating GO biological processes, molecular functions, or KEGG pathways (Fig. 5).

### Response to Stress and Hydrogen Peroxide Catabolic Process

**Figure 6:**
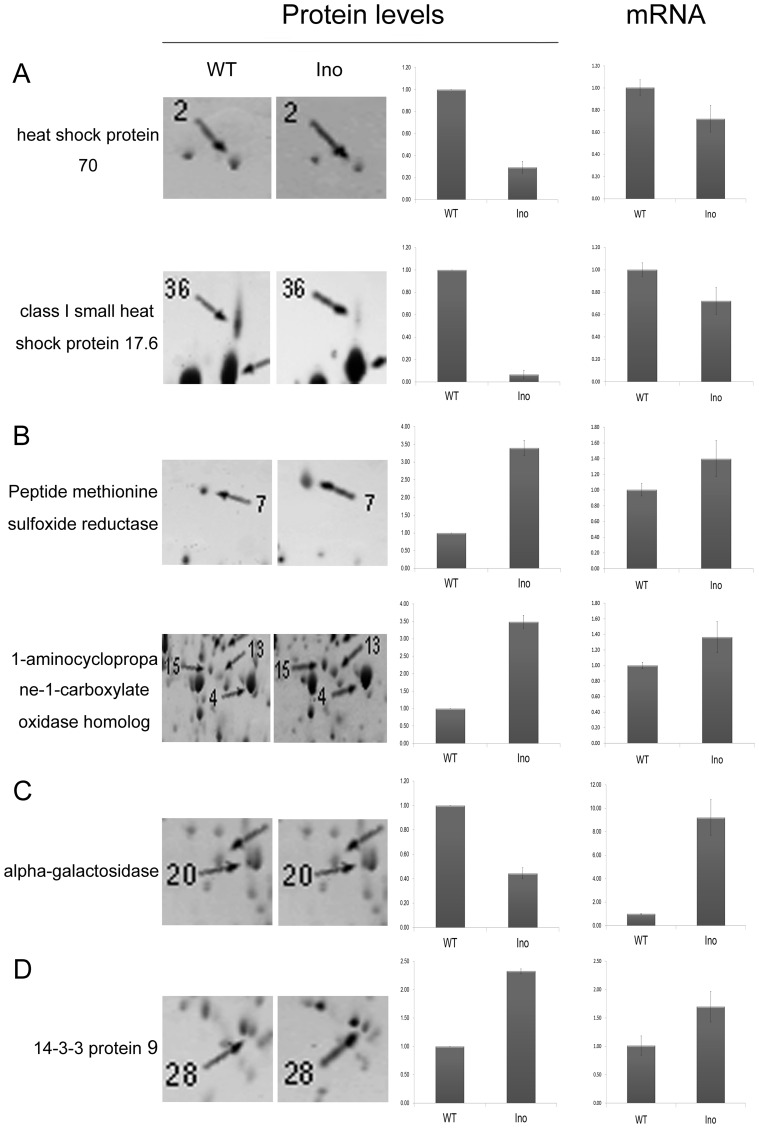
Comparison of gene expression profiles at protein and mRNA levels. Differential protein expression between *N-Ino* and *Ino* was revealed by 2D gel electrophoresis. Histograms show the changes in protein abundance. The spot number on the 2D gels and the functional annotation for each protein are shown. The mRNA expression levels were measured by quantitative real-time PCR. The gene transcript levels are indicated as fold changes after normalization against the *18* *S rRNA* gene, followed by normalization against the *N-Ino*. The results for protein and mRNA expression are means±SE from three independent experiments.

**Figure 7:**
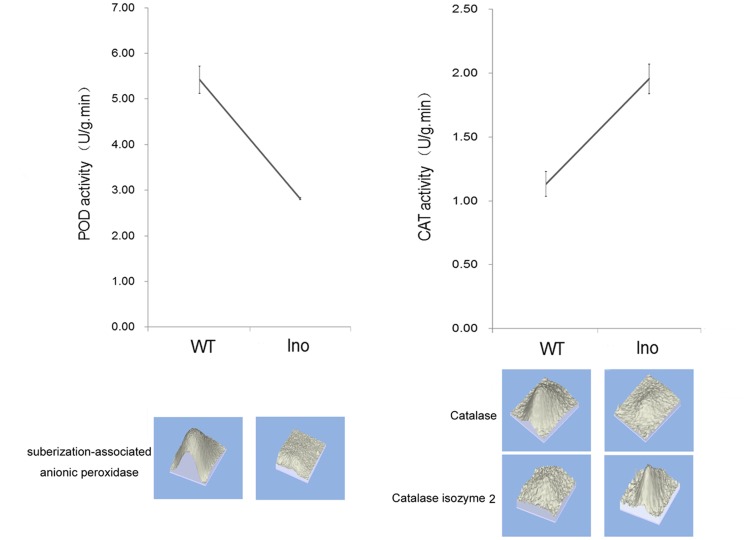
Enzyme activity of pathogen-related proteins in non-inoculated and inoculated fruit peroxidase (POD) and catalase (CAT) activity in response to *R.* *nigricans* invasion. Fruit samples were obtained from both *N-Ino* and *Ino* tomato fruit 2 days after fungal inoculation. Values presented are means±SE. The experiment was repeated three times. The corresponding proteins expression pattern is attached below the curve.

Among the identified 46 differentially expressed proteins, 9 up-regulated and 2 down-regulated proteins were involved in stress response. Heat shock proteins (HSP) have long been contemplated as intracellular chaperones that possess housekeeping and cyto-protective functions [19]. Consequently, the overexpression of HSPs was proposed as a potential therapy for various adverse situations [19]–[21]. According to UniProtKB annotations, HSP17.6, HSP17.7 and small HSP belong to the small heat shock protein (HSP20) family. As intracellular molecular chaperones, these small HSPs are involved in several defense processes, such as the refolding of denatured proteins in an ATP-independent manner [22], response to low temperatures of tomato fruit [23], and even defense to neurological diseases in human body [24]. HSP70, a 70 kDa protein belonging to the HSP70 family, has been the subject of extensive studies as it exhibits several different functions in accordance with its localization [19]. These include housekeeping functions and an essential function in promoting cell survival following stressful or harmful conditions [25]–[27]. The present study suggests that four HSPs from HSP20 and HSP70 families are involved in tomato fruit response to *R. nigricans*. The translation of HSP70 and class I small HSP17.6 proteins are reduced in the *R. nigricans* infected tissue, a trend validated by the stead state levels of their transcript (Fig. 6A),

Peroxidase (POD) is an important oxidative enzyme which catalyzes the formation of lignin and other oxidative phenols that contribute to the formation of defense barriers for reinforcing the cell structure [28]. According to the proteomic analysis and POD activity assay (Fig. 7), POD was depressed by *R. nigricans*- tomato interaction and might play a role in defense reactions against pathogen attack. Catalase (CAT), an antioxidant enzyme existing in almost all aerobically respiring organisms, is quite critical in protecting cells from the toxic effects of hydrogen peroxide [29]. Because the localization of CAT is mainly on peroxisomes, the high efficiency at reducing hydrogen peroxide may not enable it to play a central role in modulating hydrogen peroxide responses. Our proteomic data indicated that *R. nigricans* infection depressed the accumulation of catalase but induced the translation of Catalase isozyme 2. Moreover, according to Handy et al. (2012) [30], the relative contributions of glutathione peroxidases and peroxiredoxins in hydrogen peroxide reduction are dramatically greater than that of catalase. Maybe that is the reason why total CAT activity of *Ino* tomato fruit is much higher than that of the *N-Ino* fruit (Fig. 7).

### Protein Metabolic Process

Among 46 differentially expressed proteins during *R. nigricans*-tomato interaction, 4 down-regulated and 1 up-regulated proteins were involved in protein metabolism process (Fig. 4). Among which proteasome-like protein alpha subunit, RAD23-like and Skp1-like protein 3 are involved in ubiquitin-dependent protein catabolic process. The reactions and pathways in this catabolic process result in the breakdown of a protein or peptide by hydrolyzing the peptide bonds, and this is initiated by the covalent attachment of ubiquitin. The proteasome 26 S protease, a complex multiunit enzyme which shares some subunits with the proteasome or multicatalytic proteinase, is also needed in mediating the peptide hydrolysis. This characteristic is supposed to contribute to the ATP-dependent degradation and confer the ability to degrade ubiquitinated proteins [31]. Previous evidence showed that 26 S protease family is Mg^2+^-dependent ATPase and is involved in controlled protein degradation and possibly also in transcriptional regulation [32]. Degradation of protein plays essential function in all living cells. This catabolic process takes place in specialized cellular compartments, such as the lysosomes or the vacuoles in plant cells and cytosol or nucleus in all eukaryotic cells [33]. According to the cellular component information from GO, 26 S protease regulatory subunit 6A homolog and proteasome-like protein alpha subunit are located in nucleus/proteasome core complex. We might suspect that the pathogen-induced protein degradations are occurred dominantly at nucleus and proteasome complex as a result of the dramatic changed expression level of proteasome-like protein alpha subunit (3.63-fold ) (Table 1; Fig. 3A).

### Ethylene Biosynthesis

Five differentially up-regulated protein spots in infected tissue were identified to be involved in ethylene biosynthesis. Among which were methionine sulfoxide reductase (spot 7) and 1-aminocyclopropane-1-carboxylate oxidase homolog (spot 4,13,15), which is known as E4 and E8 respectively (Fig. 6B). Previous studies showed that the transcription of fruit-ripening protein E4 and E8 are coordinately activated at the onset of fruit ripening [34] and by ethylene [35]. Up to date, the induced biosynthesis of plant hormone ethylene incorporated with the response to environmental stresses and regulation of fruit ripening has been comprehensively investigated [36], [37]. From our former research work, the invasion of *R. nigricans* to tomato fruit could induce the endogenous ethylene production [1]. And almost meanwhile, the ethylene-dependent resistance pathways are launched. In the process of ethylene biosynthesis, E4 are required for the methionine metabolism [14] and take important roles during the fruit ripening. However, the regulatory mechanism is not fully understood.

Present work in our study showed that both E4 and E8 were accumulated on both protein and mRNA levels, suggesting that *R. nigricans*-invasion accelerated the ripening process of tomato fruit. Additionally, the E8 could lead to a corresponding reduction in ethylene synthesis in reverse [38]. This may explain why the ethylene production at 48 HPI displayed a downtrend when compared with that at 36 HPI [1].

### Carbohydrate Metabolic Process and Glycolysis

The six proteins that were up- (3 proteins) or down- (3 proteins) regulated after *R. nigricans* infection were involved in the process of carbohydrate metabolism. The two down-regulated proteins (chloroplast sedoheptulose-1,7-bisphosphatase and glyceraldehyde 3-phosphate dehydrogenase) are involved in the Calvin cycle. Chloroplast sedoheptulose-1,7-bisphosphatase (SBPase) is response to catalyze the dephosphorylation of sedoheptulose-1,7-bisphosphate (SBP) into sedoheptulose-1,7-phosphate (S7P) and inorganic phosphate (Pi), which is specific to the eukaryotic Calvin cycle and plays a vital role in regulating the Calvin cycle pathway [39], [40]. Besides, SBPase was reported to function in rice plants acclimation to salt stress [41] and to high temperature [42] tolerance. The glyceraldehyde-3-phosphate dehydrogenase (GAPDH) catalyzes the conversion of glyceraldehyde-3-phosphate to 1,3-bisphosphoglycerate both in cytosol and chloroplast. Munoz-Bertomeu et al. (2010) [43] have elucidated the crucial role of chloroplastic GAPDH in the control of primary metabolism in *Arabidopsis*. And GAPDH deficiency in chloroplast affects amino acid and sugar metabolism and also impairs plant development [44]. Present work indicates that the expression of SBPase and GAPDH protein in *R. nigricans* invaded tomato fruit is depressed (0.29- and 0.37-fold, respectively). Therefore, decreased catalyze activity of SBPase and the decreased amount of the key glycolytic intermediate–GAPDH [44] indicated that the Calvin cycle in *Ino* fruit was switched to defense metabolic model.

α-Galactosidase participates in many aspects of plant metabolism, such as the hydrolysis of the α-1,6 linkage of raffinose oligosaccharides during deacclimation to environment stresses. Evidence has showed that down-regulation of α-galactosidase can enhance freezing tolerance in transgenic petunia [45]. Here, our study showed that α-galactosidase protein expression is depressed by *R. nigricans* infection (0.44-fold) while the gene transcription is induced (9.22-fold) (Fig. 6C). Because of the sophisticated post-transcriptional regulation in different subcellular parts, data from translation level is not always accordant with that from transcription. Several studies have showed this inconsistent phenomenon to date. Maybe the post-transcriptional regulation is of equal importance in stress response regulation for plant. Although the expression of *α-Galactosidase* was induced by *R. nigricans*, the translation level of it is significantly depressed. One certain thing is that α-Galactosidase was involved in *R. nigricans*-tomato interaction. However, how this differential expression is regulated as a defense response and what the function of α-galactosidase is in resistance to pathogen needs further explores.

Tomato enolase and ethylene-response enolase that are involved in glycolysis process were differentially accumulated when exposed to *R. nigricans*. Enolase is a ubiquitous enzyme that catalyzes the conversion of 2-phosphoglycerate to phosphoenolpyruvate (a known plasminogen receptor in Gram-positive bacteria), which is the only dehydration step in the glycolytic pathway [46]. While ethylene-response enolase (with its gene name *ER28*), which is nearly 100% identical at the amino acid level to the tomato enolase, shows a strong specific accumulation in tomato fruit after ethylene treatment [47]. In animal, enolase can be recognized in serum from tick-infected mice, rabbits as well as Lyme disease patients [48]. And surface enolase that participates in *Borrelia burgdorferi*-Plasminogen interaction contributes to pathogen survival within feeding ticks [49]. Moreover, enolase in the outer membrane vesicles is accessible to proteolytic degradation by proteinase K [48]. However, function research focusing enolase of plant in pathogen-plant interaction is still rare. Our study showed that tomato enolase and ethylene-response enolase are up-regulated (2.36- and 2.04-fold) during the *R. nigricans*-tomato interaction, suggesting that they are involved in plant response to pathogen stress.

Synthesis, degradation, and transport of soluble carbohydrates are considered to cooperatively control their endogenous concentration in higher plants in response to environmental conditions [50], [51]. Previously, carbohydrates have been shown to have a role in freezing tolerance in cold-acclimated membranes [52], cells [53], and plants [51], [54]. Further studies on enolase and strategies to interfere with the function of enolase might contribute to the development of novel preventive measures to interrupt the infection of pathogen to plant.

### Cell Death

The 14-3-3 family of phosphoserine/phosphothreonine-binding proteins interact with various mammalian 14-3-3 isoforms [55] and play a significant role in signal transduction, particularly signaling regulated by protein kinases and phosphatases [56].

Upon previous researches, the 14-3-3 family proteins is implicated in regulating multitude of processes including cell cycle progression, regulation of apoptosis, diverse physiological and pathological processes and human disease responses [57], [58] by binding to many protein kinases and phosphatases. The expression of human 14-3-3b/a in yeast cells conferred them resistance to a variety of different stresses, including cadmium and cycloheximide, and protected against the autophagic stimuli in autophagic-deficient yeast arguing against autophagic death [59].

In plants, 14-3-3 protein has been reported to regulate key metabolic enzymes, such as nitrate reductase and sucrose synthase [60], [61], and also the activation of the plasma membrane H^+^-ATPase [52]–[64]. From the particular outcome of studies, although multiple 14-3-3 s maybe present in equal concentrations within a cell, each isoform may biochemically interact with different substrate to alter different cellular functions and localizations. Among *R. nigricans*-induced 46 differentially expressed proteins, 1 up-regulated protein (2.33-fold) was involved in cell death regulation (Fig. 6D).

Tomato 14-3-3 protein 9 is a member of 14-3-3 protein family, which is a key regulatory protein with the gene name TFT9 according to UniProtKB. In the *R. nigricans*-tomato interaction, 14-3-3 protein 9 was up-regulated, which was also confirmed by the transcript accumulation assay (Fig. 6D). This up-regulation is a response to the autophagic stimuli–*R. nigricans*, triggering the proapoptotic pathways to block nutrition accommodation against necrotrophic pathogens, preventing the development of disease and therefore protecting tomato fruit from pathogen stress.

## Conclusion

**Figure 8:**
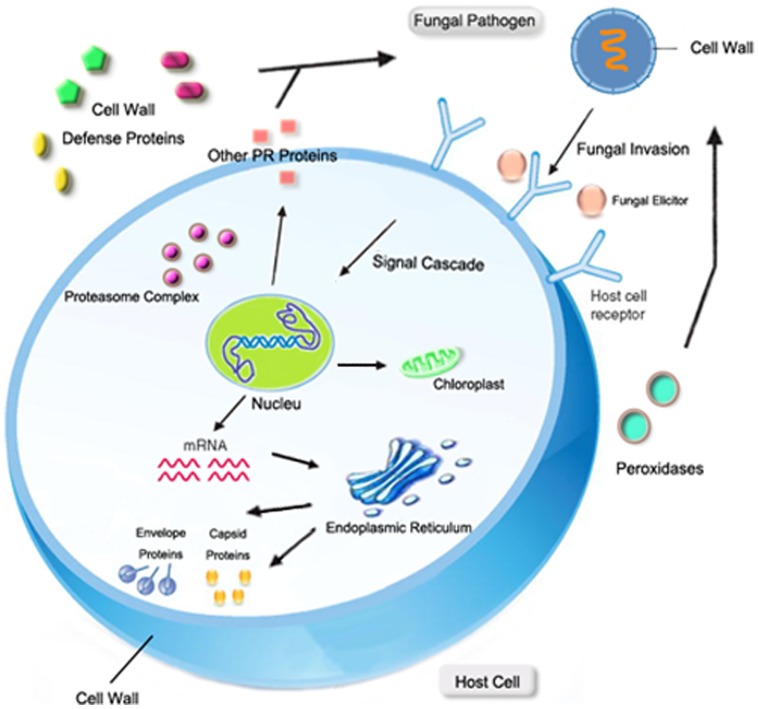
A hypothetical model representing the proteins involved in *R. nigricans*-tomato fruit interaction. Pathogen invasion is perceived by the host plant that elicits defense responses in host plant by producing proteins that degrade fungal cell wall and to overcome accompanying oxidative stress. Cell death and heat shock response occurs to prevent disease development. The chloroplast elongation factor TuB (EF-TuB) is up regulated and Calvin cycle is damaged. Viral envelope proteins are induced at host cell endoplasmic reticulum membrane. Pathogen interaction enhances ethylene production that induces the ethylene-regulated responses in pathogen. Proteases located in cytoplasm, nucleus and proteasome complex are regulated in response to pathogen attack. Organelles showed in figure are involved in the interplay between pathogen and host plant.

While encountering phytopathogenic challenge, the plant host launches various physiological and biochemical reactions to response to the invasion. A number of proteins are altered in the host during the early defense to prevent pathogen invasion, limit feeding to pathogen growth, and protect the host tissue from additional damages. The comparison of proteome analysis of the infected tissue with uninfected tissue indicated that i) *R. nigricans* invasion elicits the production of PR proteins in extracellular spaces of host tissue to degrade the organism and reduce the accompanying oxygen stress; ii) *R. nigricans* invasion propel ripening process of host MG tomato fruit; iii) *R. nigricans* invasion impact the host carbohydrate metabolic cycle and increase cell death in microenvironment; iv) protein degradation and transcription regulation are launched to response to *R. nigricans* stress. (Fig. 8).

Further characterizations of the proteome and the interactions among pathogenesis regulating changes in protein would allow developing new methods to intervene disease development. This holds promise to find new information of fundamental nature to overcome devastating losses of crop yield and postharvest storage. However, we are still far away from gaining a full understanding of plant pathogen defense mechanisms.

## Supporting Information

Figure S1 Annotated spectra for proteins identified by a single peptide or multiple peptides with each ion scored below the threshold. Spot numbers were consistent with those in 2-DE gel.
(PDF)

Table S1 Detailed sequences match searching between protein data from 2-DE and *Arabidopsis* by InParanoid.
(XLSX)
