# Correction: Unraveling the Protein Network of Tomato Fruit in Response to Necrotrophic Phytopathogenic *Rhizopus nigricans*

**DOI:** 10.1371/annotation/d93695f7-3d30-43f5-b754-ae5cf529ed3d

**Published:** 2013-10-07

**Authors:** Xiaoqi Pan, Benzhong Zhu, Yunbo Luo, Daqi Fu

Due to errors in the typesetting process, the version of Table 1 in the article was incomplete. The complete, correct version is available here: 

**Figure:**
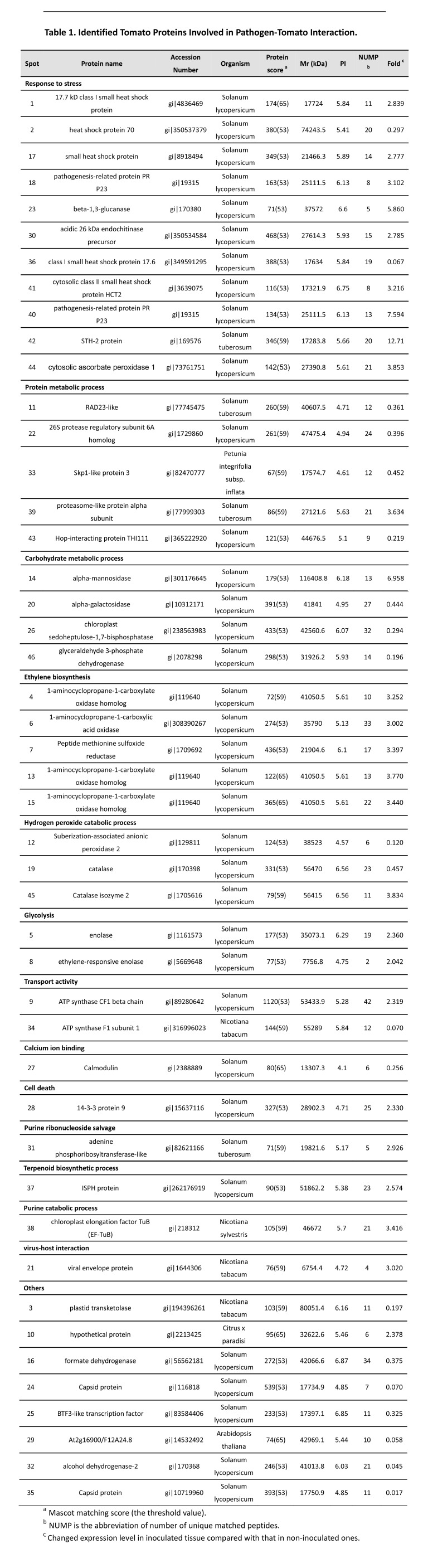


The corresponding author's email address was incorrect, and should be: daqifu@cau.edu.cn

