# Supplementary material for: Unraveling the Protein Network of Tomato Fruit in Response to Necrotrophic Phytopathogenic Rhizopus nigricans
Source: PLoS One. 2013 Sep 2;8(9):e73034. doi: 10.1371/journal.pone.0073034 (PMC3759434; doi:10.1371/journal.pone.0073034)

Supporting Figure S1. Annotated spectra for proteins identified by a single peptide or multiple peptides with each ion scored below the threshold. Spot numbers were consistent with those in 2-DE gel.

Spot number: 47

Spectra 11

Rank Protein Name	Accession No.	Protein Score	Protein Score C. I. %	Protein MW	Protein PI	Pep.Count Molecular Function
GRAS1 [Solanum lycopersicum]	gi|89474462	20	0	60504.6	5.59	8
**Protein Group**						
GRAS1 protein [Solanum lycopersicum]	gi|350537697			60504.6	5.5900 001525 8789	

Peptide Information

Calc. Mass	Obsrv. Mass	± da	± ppm	Start Seq.	End Seq.	Sequence	C. I. % Modification	Rank Result Type
1008.4567	1008.4742	0.0175	17	84	92	SPFSPQCSR		Mascot
2221.1094	2221.1577	0.0483	22	312	333	ITGVDDDESAYARGGGLQLVGK		Mascot
2230.0552	2230.261	0.2058	92	488	507	LMMAGFTQCQLSPSVGETIK	Carbamidomethyl (C)[9], Oxidation (M)[2,3]	Mascot
2426.2241	2426.2668	0.0427	18	486	507	LRLMMAGFTQCQLSPSVGETIK	Oxidation (M)[4]	Mascot
2677.2498	2677.386	0.1362	51	341	364	SCGVPFEFHGAALSGCEVQLENLR	Carbamidomethyl (C)[2,16]	Mascot
2707.3438	2707.4578	0.114	42	488	511	LMMAGFTQCQLSPSVGETIKHMLK	Carbamidomethyl (C)[9]	Mascot
2707.3438	2707.4578	0.114	42	488	511	LMMAGFTQCQLSPSVGETIKHMLK	Carbamidomethyl (C)[9]	Mascot
2943.3018	2943.6069	0.3051	104	132	159	LLGPESDTDDSCSCSLNDMVSKPSSVTR		Mascot
2943.3799	2943.3799	0.227	77	237	260	CKEPTGLELLSYMQVIFNMCPYYK	Carbamidomethyl (C)[1], Oxidation (M)[13]	Mascot

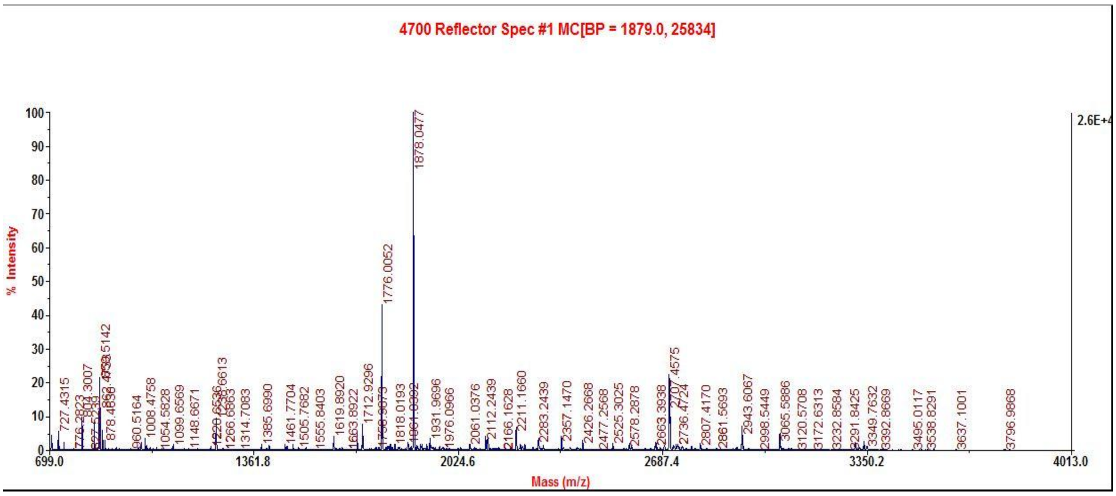

Spot number: 48

Spectra 11

Rank Protein Name	Accession No.	Protein Score	Protein Score C. I. %	Protein MW	Protein PI	Pep.Count Molecular Function
methionine sulfoxide reductase A4 [Solanum lycopersicum]	gi|344227185	24	0	28910.4	8.65	7

Peptide Information

Calc. Mass	Obsrv. Mass	± da	± ppm	Start Seq.	End Seq.	Sequence	C. I. % Modification	Rank Result Type
831.3777	831.4572	0.0795	96	248	254	GCNDPIR	Carbamidomethyl (C)[2]	Mascot
989.5302	989.4126	-0.1176	-119	45	53	TPSLYSPPK		Mascot
1663.9121	1663.6542	-0.2579	-155	1	15	MLLKTCLPSSTIATK	Carbamidomethyl (C)[6]	Mascot
1663.9121	1663.6542	-0.2579	-155	1	15	MLLKTCLPSSTIATK	Carbamidomethyl (C)[6]	Mascot
1679.9071	1679.6443	-0.2628	-156	1	15	MLLKTCLPSSTIATK	Carbamidomethyl (C)[6], Oxidation (M)[1]	Mascot
1679.9071	1679.6443	-0.2628	-156	1	15	MLLKTCLPSSTIATK	Carbamidomethyl (C)[6], Oxidation (M)[1]	Mascot
1764.8989	1764.6682	-0.2307	-131	45	59	TPSLYSPPKMSWLNK	Oxidation (M)[10]	Mascot
1981.0675	1980.8724	-0.1951	-98	5	23	TCLPSSTIATKSPLISFSK		Mascot
2580.2002	2580.1116	-0.0886	-34	174	195	QGNDVGTQYRSGIYFYTPEQEK		Mascot
2707.2568	2707.3074	0.0506	19	152	173	ECNFDTLLDVFWDRHDP		Mascot

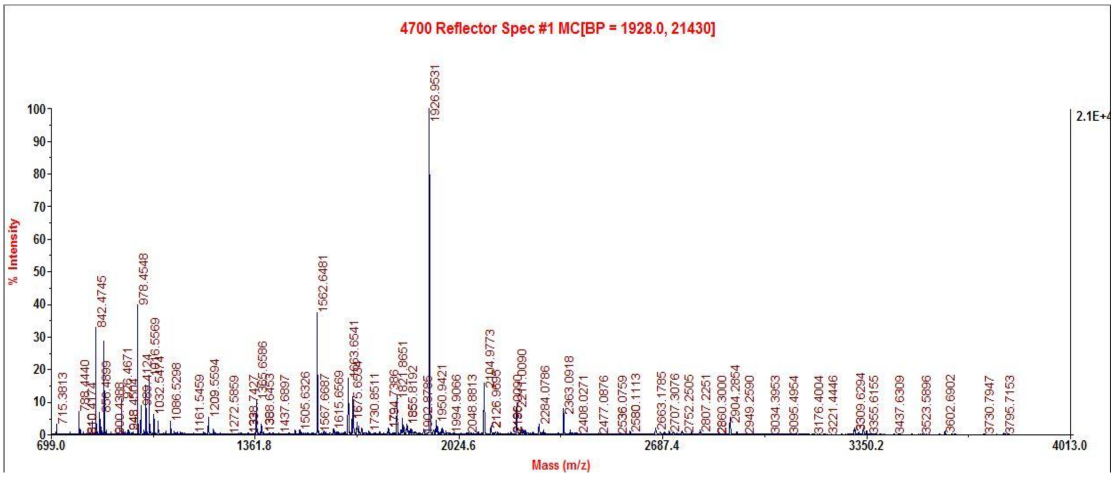

Spot number: 49

Spectra 11

Rank Protein Name	Accession No.	Protein Score	Protein Score C. I. %	Protein MW	Protein PI	Pep.Count Molecular Function
RNA-dependent RNA polymerase [Pepino mosaic virus]	gi|310006561	23	0	17231.8	9.12	6

Peptide Information

Calc. Mass	Obsrv. Mass	±da	±ppm	Start Seq.	End Seq.	Sequence	C. I. % Modification	Rank Result Type
856.5614	856.4816	-0.0798	-93	37	43	KLQNLLK		Mascot
1335.6951	1335.5599	-0.1352	-101	131	141	LHKLHQGEAMR	Oxidation (M)[10]	Mascot
1368.7052	1238.5198	-0.0624	-46	134	145	LHQGEAMRLGEK		Mascot
1812.832	1812.8308	-0.0012	-1	16	32	QVYAGDDMALDGVVSEK	Oxidation (M)[8]	Mascot
1940.927	1940.8358	-0.0912	-47	16	33	QVYAGDDMALDGVVSEKK	Oxidation (M)[8]	Mascot
2018.9316	2018.8595	-0.0721	-36	56	73	GDYAEFCGWTFTPGGIIK	Carbamidomethyl (C)[7]	Mascot

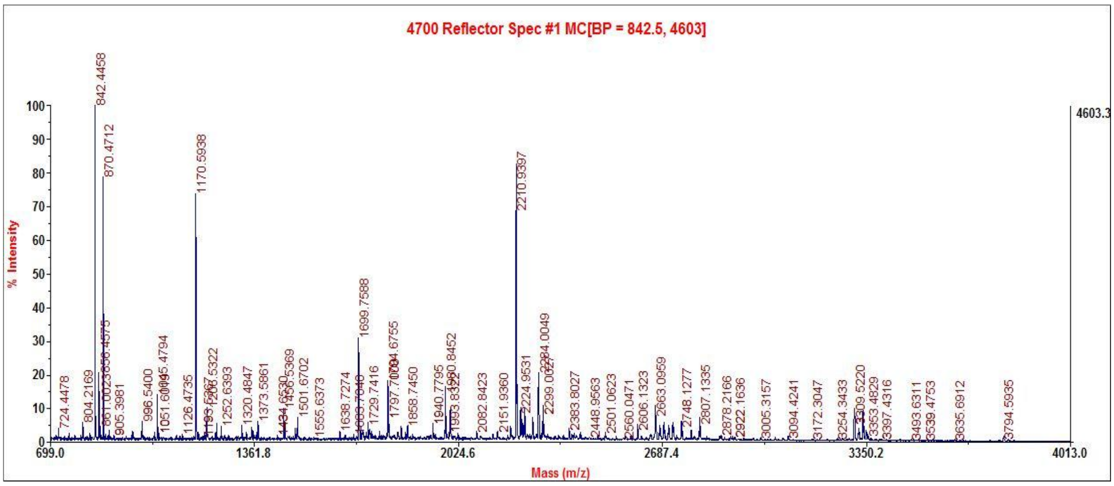

Supplement: Figure S1 — Annotated spectra for proteins identified by a single peptide or multiple peptides with each ion scored below the threshold. Spot numbers were consistent with those in 2-DE gel. (PDF) [file pone.0073034.s001.pdf]
